# Supplementary material for: Secreted Amyloid Precursor Protein Alpha (sAPPα Regulates the Cellular Proteome and Secretome of Mouse Primary Astrocytes
Source: Int J Mol Sci. 2023 Apr 12;24(8):7165. doi: 10.3390/ijms24087165 (PMC10138557; doi:10.3390/ijms24087165)
Supplement: Supplementary file 1 [file ijms-24-07165-s001.zip › Supplementary Figure and Table LegendsPeppercorn.pdf]

Figure S1

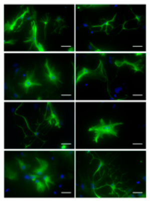

Characterization of mouse primary astrocyte cell culture showing different morphologies / subtypes of astrocytes present in primary cell culture prepared from the cortex of mouse brain. Representative images from immunocytochemical analysis of DIV21 astrocytes stained with GFAP antibody (Green) and DAPI (Blue). Scale bar 50 microm.

Figure S2

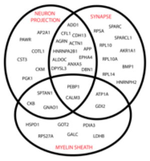

Venn Diagram showing proteins with overlapping associations. Those proteins associating with the Gene Ontology cellular compartment terms, 'synapse', 'myelin sheath' and 'neuron projection' are shown here with their gene ontology names. Refer to Table 5 for full protein names.

Supplementary Table S1

Differentially regulated proteins in the astrocyte proteome after 2 h sAPPα treatment (protein level data).

Supplementary Table S2

Differentially regulated proteins in the astrocyte proteome after 2 h sAPPα treatment (peptide level data).

Supplementary Table S3

STRING network analysis of proteins in the astrocyte proteome differentially regulated after exposure to 1 nM sAPPα for 2 h (peptide level data).

Supplementary Table S4

Differentially regulated proteins in the astrocyte secretome after 6 h sAPPα treatment (protein level data).

Supplementary Table S5

Differentially regulated proteins in the astrocyte secretome after 6 h sAPPα treatment (peptide level data).

Supplementary Table S6

STRING network analysis of proteins in the astrocyte secretome differentially regulated after exposure to 1 nM sAPPα for 6 h (peptide level data).
