# Supplementary figures and images for: Secreted Amyloid Precursor Protein Alpha (sAPPα Regulates the Cellular Proteome and Secretome of Mouse Primary Astrocytes
Source: Int J Mol Sci. 2023 Apr 12;24(8):7165. doi: 10.3390/ijms24087165 (PMC10138557; doi:10.3390/ijms24087165)

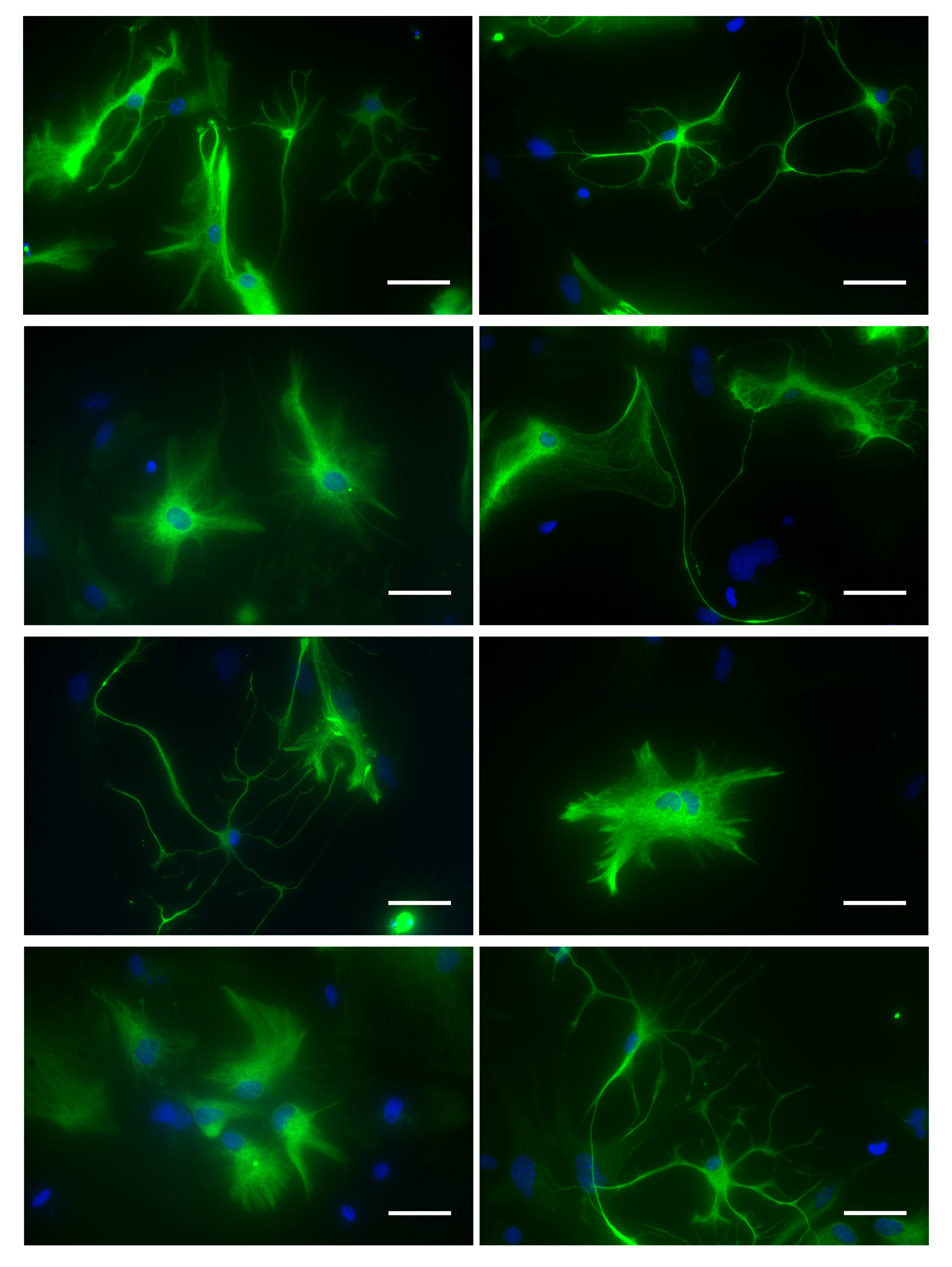

Supplement: Supplementary file 1 [file ijms-24-07165-s001.zip › Supplementary Figure S1.png]

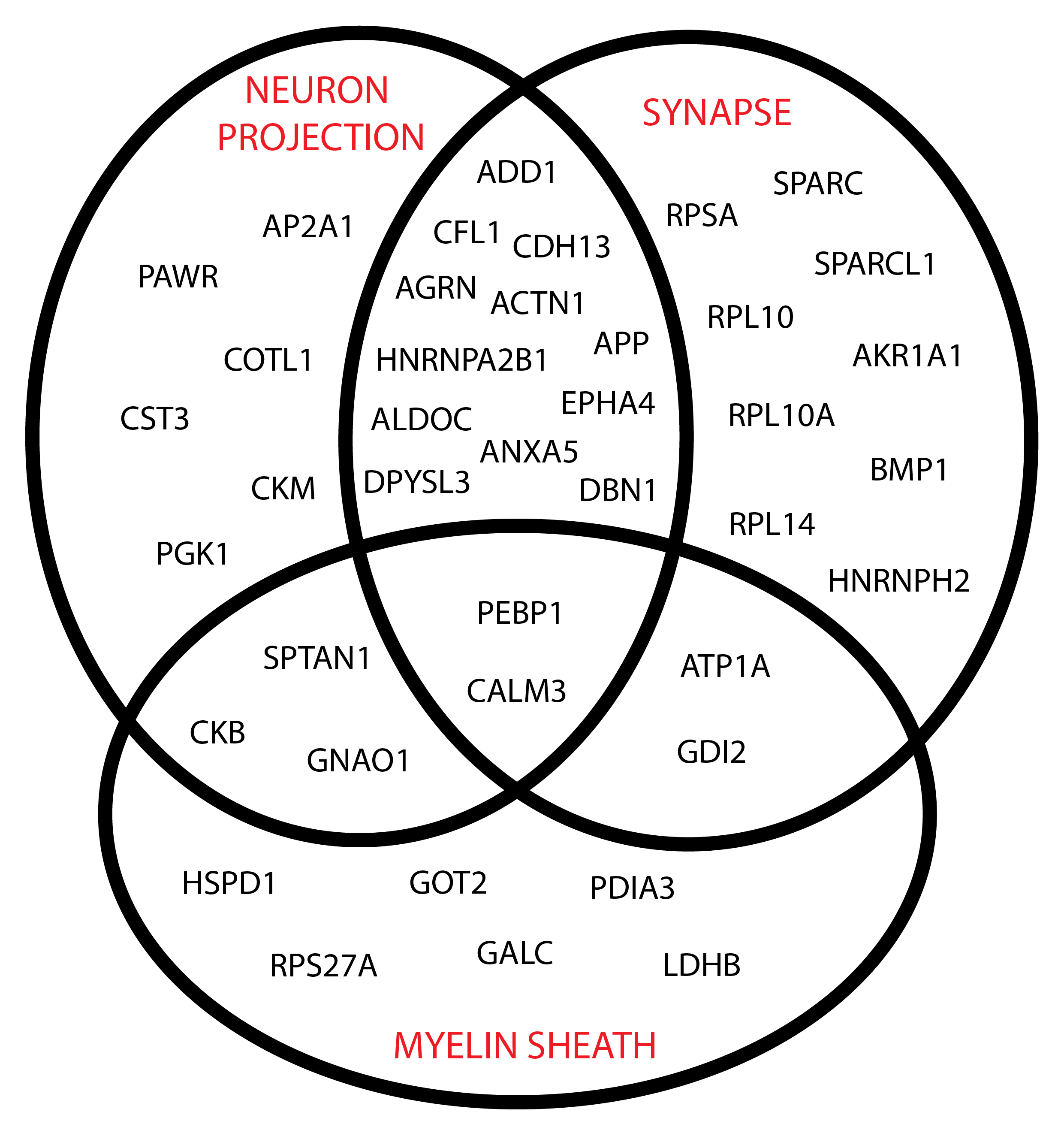

Supplement: Supplementary file 1 [file ijms-24-07165-s001.zip › Supplementary Figure S2.png]
